# Phagosomal and mitochondrial alterations in RPE may contribute to *KCNJ13* retinopathy

**DOI:** 10.1038/s41598-019-40507-8

**Published:** 2019-03-07

**Authors:** Maria Toms, Thomas Burgoyne, Dhani Tracey-White, Rose Richardson, Adam M. Dubis, Andrew R. Webster, Clare Futter, Mariya Moosajee

**Affiliations:** 1 UCL Institute of Ophthalmology, London, UK; 2 Moorfields Eye Hospital NHS Foundation Trust, London, UK; 3 Great Ormond Street Hospital for Children NHS Foundation Trust, London, UK

## Abstract

Mutations in *KCNJ13* are associated with two retinal disorders; Leber congenital amaurosis (LCA) and snowflake vitreoretinal degeneration (SVD). We examined the retina of *kcnj13* mutant zebrafish (*obelix*^*td15*^, c.502T > C p.[Phe168Leu]) to provide new insights into the pathophysiology underlying these conditions. Detailed phenotyping of *obelix*^*td15*^ fish revealed a late onset retinal degeneration at 12 months. Electron microscopy of the *obelix*^*td15*^ retinal pigment epithelium (RPE) uncovered reduced phagosome clearance and increased mitochondrial number and size prior any signs of retinal degeneration. Melanosome distribution was also affected in dark-adapted 12-month *obelix*^*td15*^ fish. At 6 and 12 months, ATP levels were found to be reduced along with increased expression of glial fibrillary acidic protein and heat shock protein 60. Quantitative RT-PCR of *polg2*, *fis1*, *opa1*, *sod1/2* and *bcl2a* from isolated retina showed expression changes consistent with altered mitochondrial activity and retinal stress. We propose that the retinal disease in this model is primarily a failure of phagosome physiology with a secondary mitochondrial dysfunction. Our findings suggest that alterations in the RPE and photoreceptor cellular organelles may contribute to *KCNJ13*-related retinal degeneration and provide a therapeutic target.

## Introduction

*KCNJ13* (MIM #603208) is a three-exon gene located on chromosome 2q37 which encodes the 360-amino acid protein Kir7.1, a low-conductance inwardly rectifying potassium channel (Kir) that functions as a homotetramer^1–4^. Kir7.1 is localized at the plasma membrane of a variety of ion-transporting epithelia, including the retinal pigment epithelium (RPE)^5–7^, a cell monolayer essential for photoreceptor function and survival^8^. Mutations in *KCNJ13* have been linked with two ocular disorders; (i) autosomal recessive Leber congenital amaurosis (LCA, MIM #614186), a severe early onset retinal dystrophy with RPE and photoreceptor loss causing blindness from birth^9–11^, and (ii) autosomal dominant snowflake vitreoretinal degeneration (SVD, MIM #193230), a disorder characterized by a fibrillar vitreous degeneration and crystalline-like deposits in the retina^6^.

The Kir7.1 channel is expressed in a range of tissues, including the intestine, kidney, retina and RPE^2,3,6,7,12^. In the RPE, Kir7.1 is localized to the apical membrane at the interface with the photoreceptor outer segments, where it facilitates potassium ion (K^+^) efflux to the subretinal space in order to offset a decrease in levels in response to light exposure^13,14^. Additionally, K^+^ transport provides the driving force for controlled fluid flow across the blood–retina barrier formed by the RPE^3,15^. Kir7.1 shows co-localization with the Na^+^/K^+^ pump, suggesting that it is involved in K^+^ recycling required to keep up with high rates of epithelial ion transport^12^.

*Kcnj13* mouse models have been independently generated to examine Kir7.1 function in disease. Homozygous *Kcnj13* null mutant mice showed cleft palate and moderate retardation in lung development, suffering early postnatal mortality by P0^16^. The retinal phenotype has been examined in *Kcnj13* mosaic mice^17^ and most recently in conditional knockout mice generated using CRISPR/Cas9, where loss of *Kcnj13* expression in the RPE caused severe and progressive thinning of the outer nuclear layer from 15 days post birth and a reduced response to light^18^. These findings highlight the essential role of RPE-based Kir7.1 in retinal photoreceptor function and survival.

The *obelix* (*obe*^*td15*^) zebrafish mutant, generated through ENU mutagenesis, harbors a missense mutation, c.502T > C, p.(Phe168Leu) in *kcnj13*, which affects the transmembrane region abolishing K^+^ conductance by disrupting K^+^ permeation through the channel^19^. These zebrafish show a defect in skin pattern formation, displaying broader stripes than wild-type fish; the RPE or retina were not investigated. In this mutant, the skin melanophores were not able to respond correctly to the melanosome dispersion signal derived from the sympathetic neurons and this resulted in aberrant melanosome aggregation. In view of these findings, we have characterized the retinal degeneration in the homozygous *obe*^*td15*^ zebrafish, identifying alterations in melanosome function with phagosome and mitochondrial activity linked to retinal stress, furthering our understanding of the pathophysiology associated with *KCNJ13* in the retina.

## Results

### Retinal morphology and visual function of *obe*^*td15*^ zebrafish

**Figure 1:**
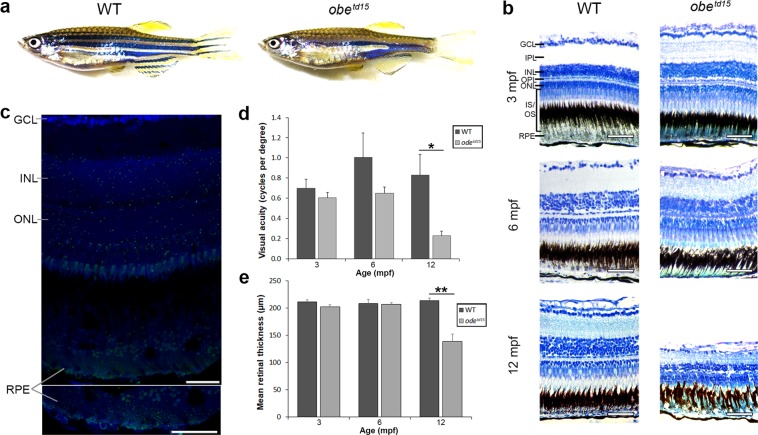
Retinal structure and function in *obe*^*td15*^ zebrafish. (**a**) Wholemount morphology of adult wild-type (WT) and *obe*^*td15*^ zebrafish. (**b**) Retinal histology of *obe*^*td15*^ zebrafish at 3, 6 and 12 months post fertilization (mpf). (**c**) Expression of *kcnj13* mRNA (green) in the WT adult zebrafish retina detected using an RNAscope assay. Sections are counterstained with DAPI nucleic acid stain (blue). (**d**) Visual acuity (cycles per degree) of *obe*^*td15*^ zebrafish at 3, 6 and 12 mpf, measured using optokinetic response assay (minimum n = 4, mean ± SEM). (**e**) Retinal thickness (µm) of *obe*^*td15*^ zebrafish at 3, 6 and 12 mpf, measured using OCT (n = 5 for each age, mean ± SEM). GCL, ganglion cell layer; IPL, inner plexiform layer; INL, inner nuclear layer; OPL, outer plexiform layer; ONL, outer nuclear layer; IS/OS, outer and inner segments; RPE, retinal pigment epithelium. *p < 0.05, **p < 0.01. Scale bars = 50 μm (**b**) and 25 μm (**c**).

The wholemount morphology of the homozygous *obe*^*td15*^ zebrafish was unremarkable until 1 month post fertilization (mpf), when the characteristic broader stripe skin pigmentation was noted (Fig. 1a). There were no gross ocular morphological differences between wild-type AB (WT) and *obe*^*td15*^ zebrafish at any timepoint. To determine spatial gene expression of *kcnj13* within the WT adult zebrafish retina, fluorescent *in situ* hybridization using the RNAscope assay was carried out on retinal cryosections (Fig. 1c). Individual *kcnj13* mRNA transcripts were visualized as spots of fluorescence throughout the inner and outer retina, distributed evenly through the ganglion cell layer, inner nuclear layer, outer plexiform layer, outer nuclear/photoreceptor layer and RPE. *odc1* and *dapB* (bacterial gene) probes were used as positive and negative controls, respectively (Supplementary Fig. S1). The *dapB* probe showed little or no fluorescence, corresponding to absent gene expression.

The retinal histology of *obe*^*td15*^ was comparable to WT fish until 12 mpf, at which point the mutant retina showed extensive retinal degeneration with disruption of the photoreceptor layer and RPE, with overall retinal thinning (Fig. 1b). In order to determine the visual function of *obe*^*td15*^ zebrafish, optokinetic response testing was undertaken at 3, 6, and 12 mpf comparing WT, *obe*^*td15*^ and positive control WT fish injected with ouabain to induce a chemical retinal degeneration. The results show that the *obe*^*td15*^ zebrafish had a similar visual acuity to WT fish at 3 mpf (0.70 ± 0.09 cycles per degree [cpd] in WT, 0.61 ± 0.05 cpd in mutant fish) but at 6 mpf WT fish showed a greater but not significant increase in acuity (1.0 ± 0.24 cpd in WT, 0.65 ± 0.06 cpd in mutant fish) (Fig. 1d). At 12 mpf, mean visual acuity of *obe*^*td15*^ zebrafish showed a significant decline and was measured as 0.23 ± 0.04 cpd compared to 0.83 ± 0.21 cpd in age-matched WT (p < 0.05). A minimum of four WT and *obe*^*td15*^ zebrafish were assessed per timepoint. Optokinetic responses were not observed in ouabain-injected fish.

**Figure 2:**
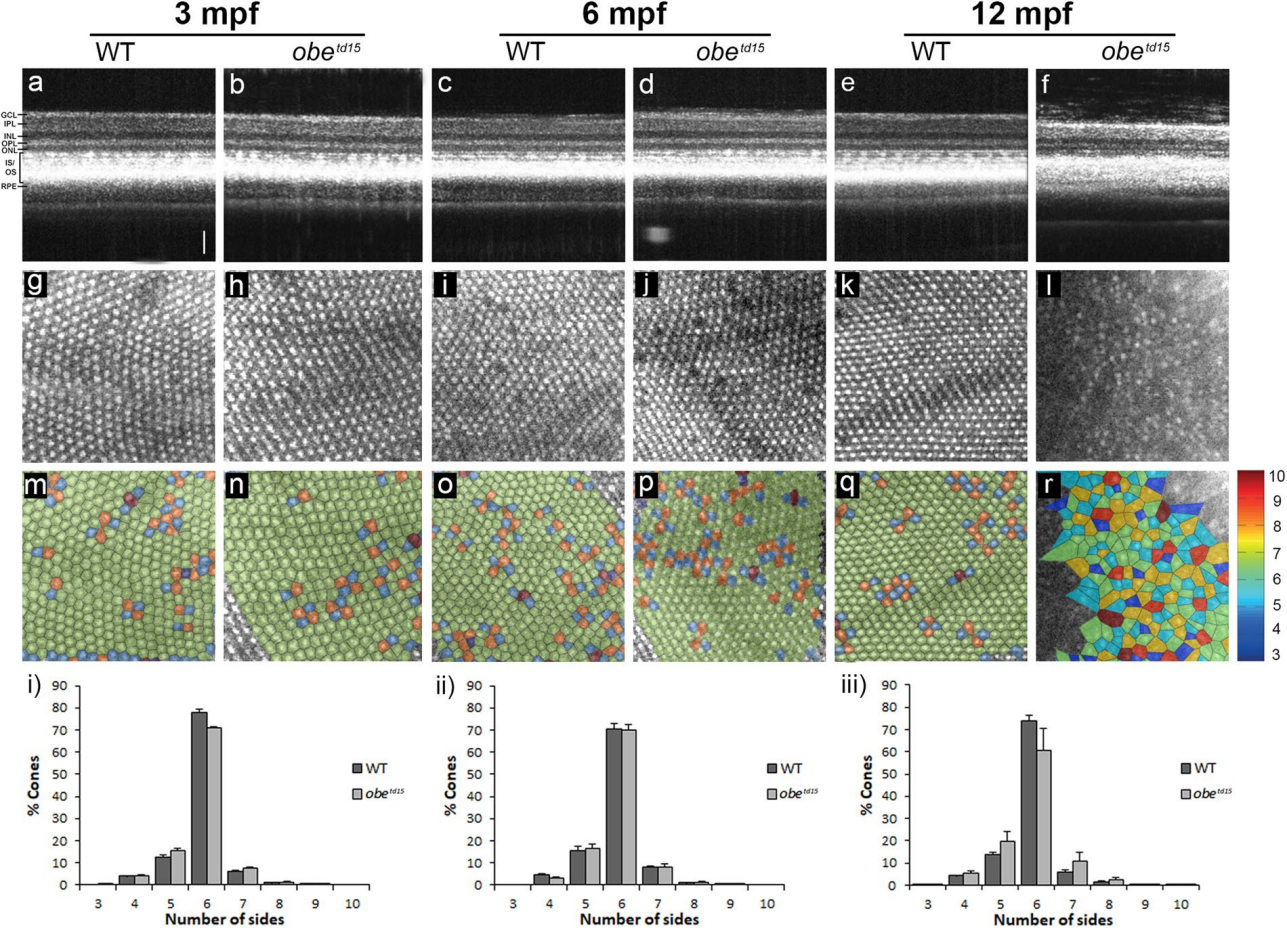
Cone photoreceptor mosaic in *obe*^*td15*^ zebrafish. Optical coherence tomography was used at 3, 6 and 12 months post fertilization (mpf) to examine retinal cross-sectional views (**a–f**), *en face* cone photoreceptor mosaics (**g–l**) and corresponding Voronoi domain overlays (**m–r**) in which a Voronoi polygon is associated with each cone photoreceptor and color-coded according to the number of sides it possesses (color key shown on right). Bar charts showing the mean percentage of 3- to 10-sided Voronoi domains in wild-type (WT) and *obe*^*td15*^ zebrafish at (i) 3 mpf, (ii) 6 mpf and (iii) 12 mpf (n = 5 for each age, mean ± SEM). GCL, ganglion cell layer; IPL, inner plexiform layer; INL, inner nuclear layer; OPL, outer plexiform layer; ONL, outer nuclear layer; PR, photoreceptors; RPE, retinal pigment epithelium. Scale bar = 50 μm.

Retinal structure was further examined using spectral domain optical coherence tomography (OCT), a non-invasive imaging technique based on interferometry that enables visualization of various retinal features *in vivo*^20^. Cross-sectional B-scan images of WT and *obe*^*td15*^ confirmed histological changes showing that significant retinal thinning and loss of photoreceptor layers are apparent at 12 mpf (Fig. 2a–f). Measurement of retinal thickness from OCT images (n = 5) revealed that *obe*^*td15*^ retinas were similar in thickness at 3 and 6 mpf, while mean thickness at 12 mpf was significantly reduced at 139 ± 13.5 µm compared to 214 ± 4.5 µm in WT (p < 0.05) (Fig. 1e).

To assess the regularity of the *en face* ultraviolet cone mosaic images (Fig. 2g–l) Voronoi domain analysis was used, revealing loss of the regular triangular lattice of 6-sided polygons in *obe*^*td15*^ retinas at 12 mpf (Fig. 2m–r). At 3, 6 and 12 mpf, the mean percentage of cones with 6 neighbours in the *obe*^*td15*^ fish was 70.8 ± 0.9%, 69.9 ± 2.6% and 60.6 ± 8.9% respectively, compared to WT fish at the corresponding age; 77.8 ± 1.7%, 70.3 ± 2.5% and 74.1 ± 2.2% respectively (n = 5) (Fig. 2i–iii). It should be noted that several retinas were too severely affected to be included in the analysis due to an inability to distinguish the cones.

### Retinal ultrastructure of *obe*^*td15*^ zebrafish

**Figure 3:**
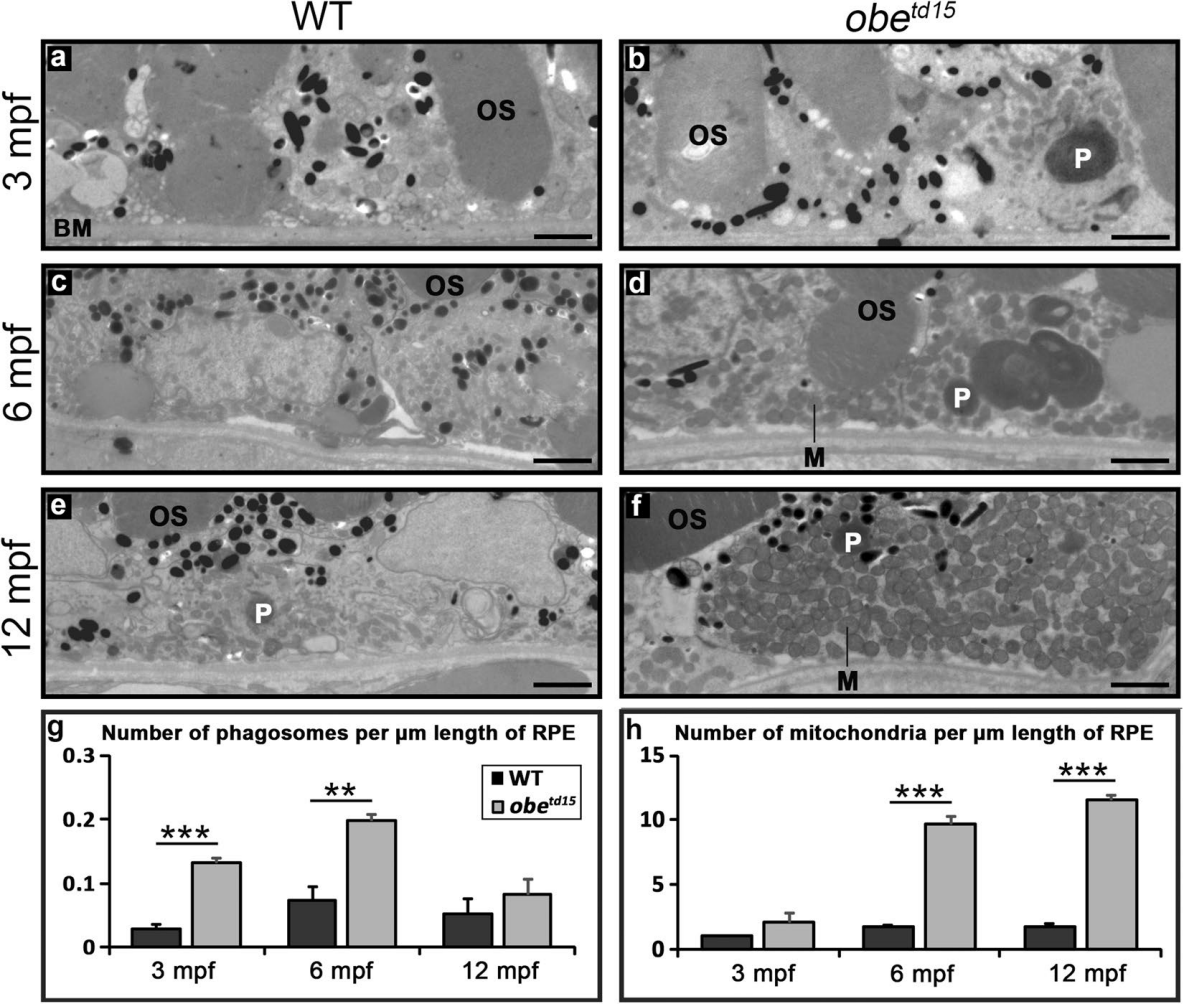
Increased mitochondrial abundance and altered phagosome clearance in the retinal pigment epithelium (RPE) of *obe*^*td15*^ zebrafish. TEM cross-sections of the RPE show little difference at 3 months post fertilization (mpf) (**a,b**), while at 6 mpf (**c,d**) and 12 mpf (**e,f**) there is a significant increase in the number of mitochondria (M) in *obe*^*td15*^ compared to wild-type (WT) eyes. Additionally, there is a significant increase in the number of phagosomes (P) in *obe*^*td15*^ eyes at 3 mpf and 6 mpf. Bar charts displaying numbers of phagosomes and mitochondria per µm length of RPE are shown in (**g,h**). OS, photoreceptor outer segments; BM, Bruch’s membrane. Results are mean ± SEM (n = 3). **p < 0.01, ***p < 0.001. Scale bars = 2 µm.

Retinal ultrastructure was examined at 3, 6 and 12 mpf using transmission electron microscopy (TEM) (Fig. 3). Numbers of mitochondria and phagosomes in the RPE were assessed, the latter of which are vesicles containing shed photoreceptor outer segments for degradation. At 3 mpf, a modest increase in mitochondria per µm was noted in the RPE of *obe*^*td15*^ zebrafish, with a substantial 4.3-fold increase in phagosomes, indicative of altered phagosome clearance (n = 3) (p < 0.001). At 6 mpf, there was a 5.5-fold increase in mitochondria (p < 0.001) and a 2.8-fold increase in phagosomes in the mutant RPE compared to WT (p < 0.01). At 12 mpf, the mitochondria numbers remained high with a 6.4-fold increase (p < 0.001), but the phagosome number declined to 1.6-fold, with no significant difference between *obe*^*td15*^ and WT fish at this point. The expansion in numbers of mitochondria and phagosomes in the *obe*^*td15*^ retinas resulted in a displacement of the cellular organelles from the basal to the apical site of the RPE. The *obe*^*td15*^ 12 mpf retinas exhibited areas of severe disease containing disordered photoreceptor outer segments with islands of preserved tissue (Supplementary Fig. S2). This is similar to the pattern of degeneration commonly seen in human retinal dystrophies and further long-term studies would reveal the late natural history.

**Figure 4:**
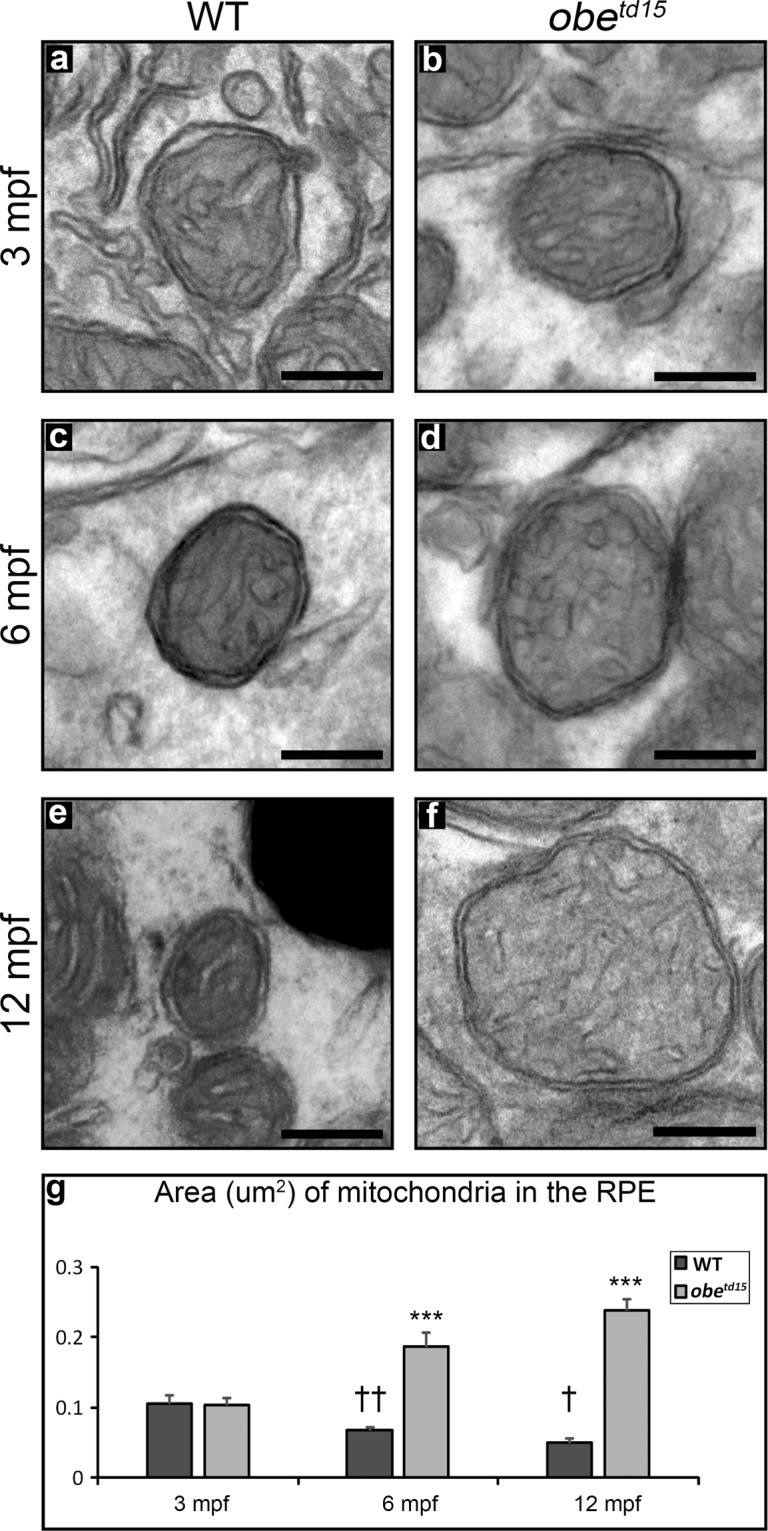
Increased mitochondrial size in the retinal pigment epithelium (RPE) of *obe*^*td15*^ zebrafish. Mitochondrial size in the wild-type (WT) RPE at (**a**) 3 months post-fertilisation (mpf), (**c**) 6 mpf and (**e**) 12 mpf shows a decrease with age, while mitochondrial size in the *obe*^*td15*^ RPE at (**b**) 3 mpf, (**d**) 6 mpf and (**f**) 12 mpf shows an increase with age. Mitochondrial area measured from TEM images displayed on bar chart (**g**). Results are mean ± SEM. 13 mitochondria measured per WT and mutant age group. Statistical significance relative to WT of different ages ^†^p < 0.05, ^††^p < 0.01. Statistical significance relative to WT against *obe*^*td15*^ of the same age. ***p < 0.001. Scale bar = 200 nm.

Measurement of mitochondrial size revealed a progressive increase over time in the *obe*^*td15*^ zebrafish RPE, with a mean area of 0.10 ± 0.008 um^2^, 0.19 ± 0.018 um^2^ and 0.24 ± 0.015 um^2^ at 3, 6 and 12 mpf respectively (13 mitochondria measured per WT and mutant age group) (Fig. 4). Mitochondria in the WT RPE were similar in size to the mutant at 3 mpf, and showed an overall decrease in size with age to 0.07 ± 0.004 um^2^ and 0.05 ± 0.006 um^2^ at 6 and 12 mpf respectively. The *obe*^*td15*^ mitochondria were significantly larger than WT at these timepoints, with a 4.9-fold increase in mean area compared to WT at 12 mpf (p < 0.001).

**Figure 5:**
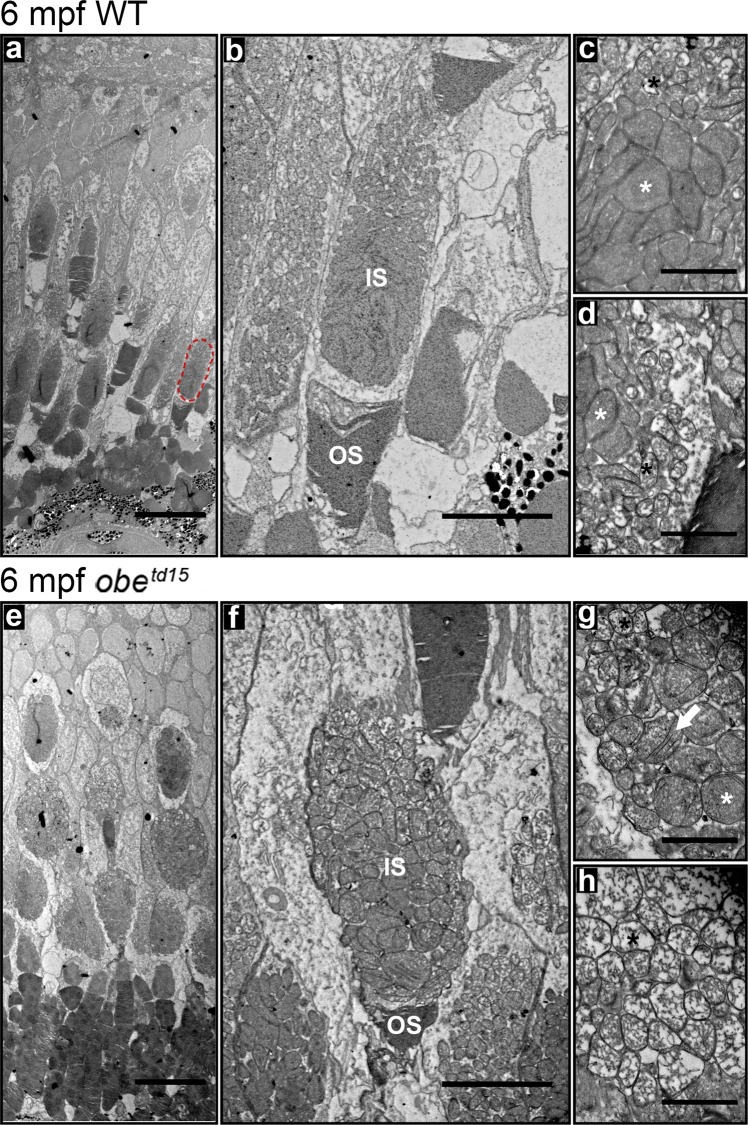
Altered mitochondrial morphology in the *obe*^*td15*^ cone photoreceptors. Ultrastructural examination of the cone photoreceptors in 6 months post fertilization (mpf) wild-type (WT) (**a–d**) and *obe*^*td15*^ fish (**e–h**). A red-green cone inner segment is highlighted on (**a**) (red dotted circle). Higher magnification images showed an altered morphology of the mitochondria in the *obe*^*td15*^ red-green cone inner segments (**f–h**). Electron-lucent mitochondria (black asterisks) were enlarged and electron-dense mitochondria (white asterisks) were rounder and less closely packed. The electron-dense mitochondria had juxtaposition of the cristae membranes (white arrow). IS, inner segments; OS, outer segments. Scale bars = 20 µm (**a,e**), 5 µm (**b,f**) and 1 µm (**c,d,g,h**).

The mitochondria in the inner segment ellipsoid of the double cone (red-green) photoreceptors were also examined in the *obe*^*td15*^ retina. At 6 mpf, both electron-lucent and electron-dense mitochondria were present in both WT and mutant fish (Fig. 5). However, in the *obe*^*td15*^ retina, the electron-lucent mitochondria appeared enlarged (Fig. 5h), and the electron-dense mitochondria were rounder and less closely packed, displaying a juxtaposed linearization of the cristae membranes that appear as narrow structures that have high contrast in the electron microscope (Fig. 5g).

**Figure 6:**
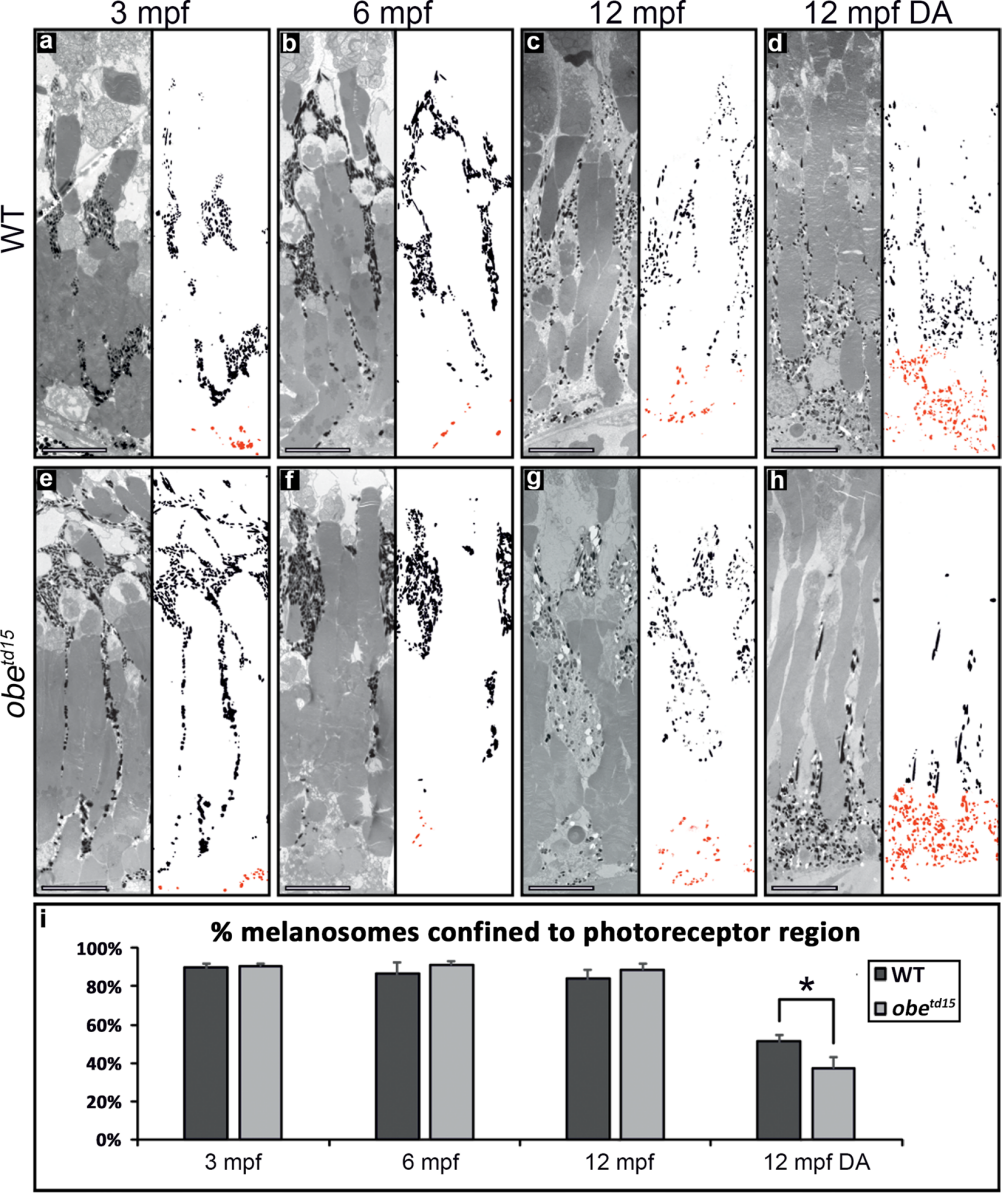
Melanosome localization in the *obe*^*td15*^ retinal pigment epithelium (RPE) in normal and dark-adapted conditions. TEM images of wild-type (WT) (**a–d**) and *obe*^*td15*^ (**e–h**) RPE at 3 months post fertilization (mpf), 6 mpf and 12 mpf with neighbouring panels showing digitally extracted melanosomes with the basal RPE-localized melanosomes false-colored in red. Using these extracted melanosomes, the proportion of melanosomes localized to the photoreceptor region was calculated, shown in bar chart (**i**). There was little difference in the proportion of melanosomes localised to the apical photoreceptor region of the RPE in zebrafish exposed to a normal daily light cycle. Whereas, dark-adaptation (DA) of 12 mpf *obe*^*td15*^ zebrafish (**h**) caused significantly more melanosomes to localize to the basal region of the RPE compared to WT zebrafish eyes (**d**). Results are the mean (from three regions from three eyes) ± SEM. *p < 0.05. Scale bars = 10 µm.

In fish, the melanosomes of the RPE exhibit a redistribution from the basal cell body into the apical processes upon the onset of light, which is reversed in the dark^21^. Examination of melanosome distribution in the RPE at 3, 6 and 12 mpf in fully light-adapted fish exposed to normal light:dark cycle did not reveal any significant differences in the percentage of melanosomes localized to the apical photoreceptor region between the WT and *obe*^*td15*^ RPE (Fig. 6). In order to further assess melanosome function, 12 mpf WT and mutant fish were dark-adapted for 48 hours before collection. This revealed a significantly increased basal aggregation in the RPE of *obe*^*td15*^ zebrafish compared to a more apical localization in the WT (p < 0.05).

### Mitochondrial biogenesis and metabolism

**Figure 7:**
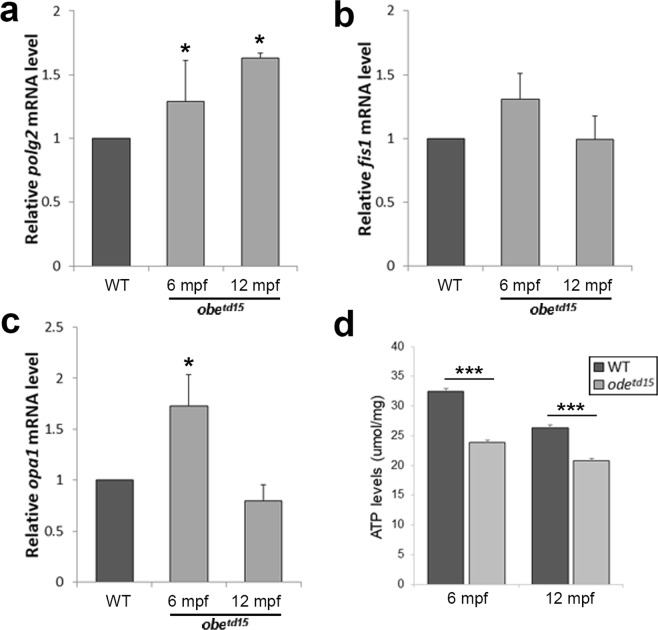
Mitochondrial biogenesis and ATP levels in the *obe*^*td15*^ retina. Quantitative RT-PCR was used to determine relative mRNA expression levels of *polg2* (**a**), *fis1* (**b**), and *opa1* (**c**) at 6 and 12 months post fertilization (mpf) (n = 3 for each age, mean ± SEM). (**f**) ATP levels were examined using a luciferin-luciferase assay in the *obe*^*td15*^ retina at 6 and 12 mpf (n = 5 for each age, mean ± SEM). *p < 0.05, ***p < 0.001.

In order to verify the qualitative ultrastructural appearances of the mitochondrial changes in the RPE, we conducted qRT-PCR of *polg2* (mitochondrial DNA polymerase subunit gene), *fis1* (fission gene), *opa1* (fusion gene) in isolated retinal and RPE tissue of WT and *obe*^*td15*^ zebrafish at 6 and 12 mpf (n = 3) (Fig. 7a–c). *polg2* expression was upregulated in *obe*^*td15*^ tissue compared to WT, showing 1.29 ± 0.32 fold and 1.62 ± 0.04 fold increases at 6 and 12 mpf respectively (p < 0.01). *fis1* was elevated at 6 mpf with a 1.31 ± 0.31 fold increase in expression before declining at 12 mpf to a 0.99 ± 0.31 fold change in the mutant compared to WT. A similar trend was observed in *opa1* expression which showed a 1.73 ± 0.31 fold increase at 6 mpf (p < 0.01) before dropping to levels lower than WT at 12 mpf (0.80 ± 0.16 fold change). An ATP assay was performed at 6 and 12 mpf (n = 5) (Fig. 7d). This revealed a 26.2% and a 21.2% reduction in ATP levels at 6 and 12 mpf respectively in the mutant relative to WT (p < 0.001 and p < 0.001). We investigated mitochondrial function in the 6 mpf *obe*^*td15*^ retina using Seahorse XF analysis (see Supplementary Fig. S3), which revealed a decrease in baseline oxygen consumption rate (OCR) in the mutant, but did not reach statistical significance (n = 3, p = 0.0558).

### Retinal stress

#### Müller cell activation

**Figure 8:**
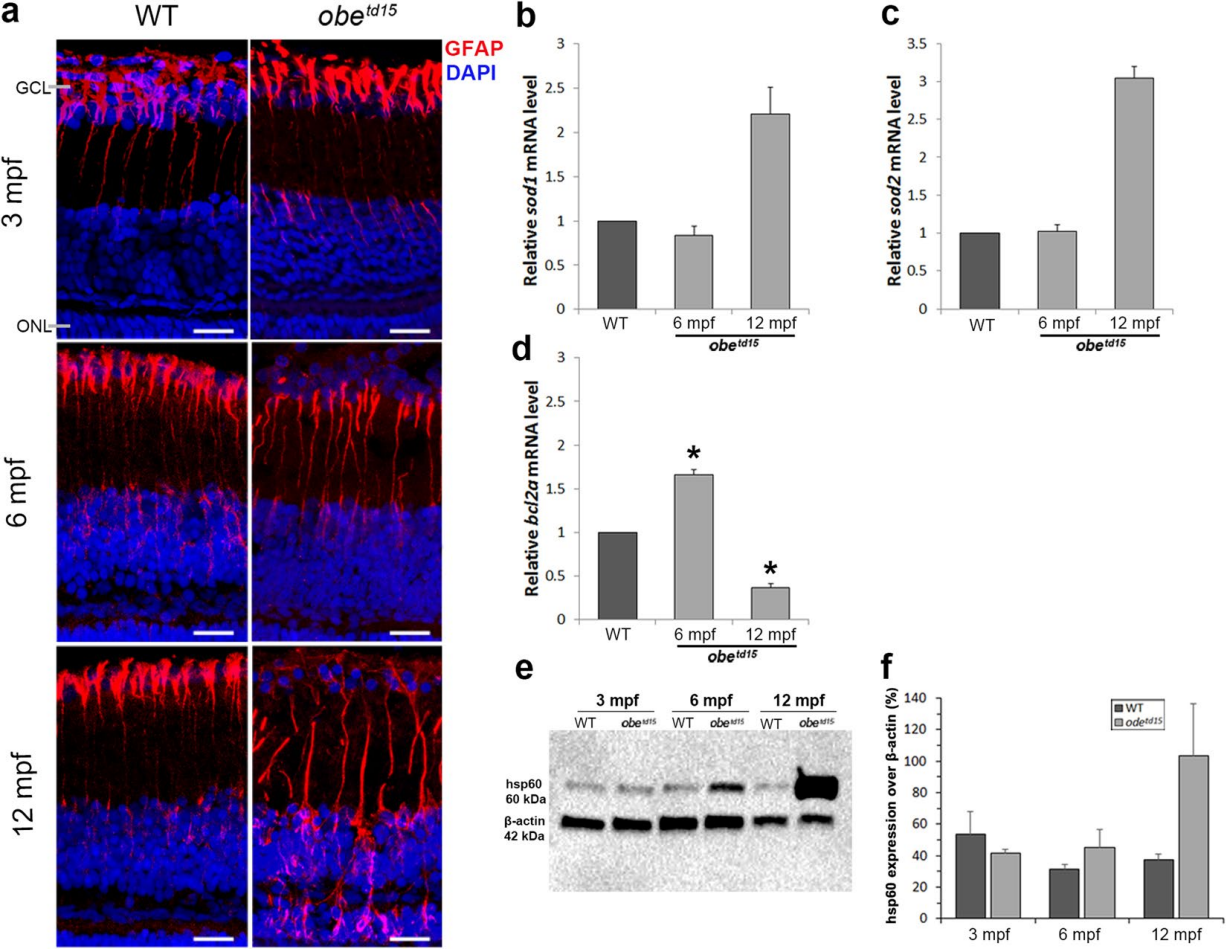
Retinal stress in *obe*^*td15*^ zebrafish. Immunostaining of the retina at 3, 6 and 12 mpf using anti-ZRF1 to detect expression of glial fibrillary acidic protein (red), counterstained with DAPI nucleic acid stain (blue) (**a**). Quantitative RT-PCR was used to determine relative mRNA expression levels of *sod1* (**b**), *sod2* (**c**) and *bcl2a* (**d**) genes at 6 and 12 mpf (n = 3 for each age, mean ± SEM). Western blot analysis of heat shock protein 60 (hsp60) expression (**e**). The blot was re-probed with anti-β-actin as a loading control for samples. Bands of interest are cropped from full-length blot presented in Supplementary Fig. S4. The bar chart (**f**) shows mean ± SEM result from three independent experiments. *p < 0.05. Scale bar = 50 µm.

The glial fibrillary acidic protein (GFAP) is known to be upregulated by glial cells in response to retinal injury or stress^22^. Immunostaining of GFAP (detected using anti-ZRF1) showed expression in the Müller cell endfeet and processes in both WT and mutant retinas (Fig. 8a). By 6 mpf, GFAP expression became increased further along the processes in the *obe*^*td15*^ retina and at 12 mpf expression was irregular and extended into the outer nuclear layer. The WT retina maintained a similar expression pattern at all ages.

Levels of apoptotic cell death in the retina at 6 and 12 mpf were examined using a TUNEL assay (ApopTag® Plus Fluorescein *In Situ* Apoptosis Detection Kit, Merck Millipore). We were unable to detect notable levels in the mutant at either age (data not shown). This suggests that significant degeneration occurs rapidly between these time points and the majority of apoptosis is complete at 12 mpf when widespread destruction is apparent.

#### Mitochondrial stress

To examine levels of mitochondrial stress in the *obe*^*td15*^ RPE and retina, we carried out qRT-PCR of *bcl2a, sod1* and *sod2* at 6 and 12 mpf (n = 3) (Fig. 8b–d). The *sod1* and *sod2* genes encode cytoplasmic and mitochondrial superoxide dismutases respectively, which are antioxidant enzymes involved in oxidative stress response^23^. At 6 mpf, expression of *sod1* and *sod2* were similar to age-matched WT, showing 0.84 ± 0.10 fold and 1.03 ± 0.08 fold changes respectively. Levels of both genes were elevated at 12 mpf, with a 2.21 ± 0.08 fold increase in *sod1* and a 3.04 ± 0.16 fold increase in *sod2* expression in *obe*^*td15*^ fish versus WT. qRT-PCR of *bcl2a*, an apoptosis regulator in the mitochondrial death pathway, showed that expression was increased 1.66 ± 0.06 fold at 6 mpf in the mutant versus WT (p < 0.05), before showing a decline to 0.36 ± 0.05 fold at 12 mpf (p < 0.05).

To further investigate mitochondrial stress, a western blot assay was carried out for hsp60, a mitochondrial chaperone involved in stress response^24^ (Fig. 8e,f). This revealed comparable levels of protein expression at 3 mpf in WT and *obe*^*td15*^ RPE and retinal tissue. At 6 and 12 mpf, hsp60 was increased in the mutant by 13.7% and 66.3% respectively.

## Discussion

The Kir7.1 protein is an inwardly rectifying potassium channel linked to two forms of retinal dystrophy, LCA and SVD. Previous mouse work demonstrated the essential function of Kir7.1 in the RPE but was limited by the early lethality of *Kcnj13* homozygous mice^17^. We explored the retinal disease pathology using the *obe*^*td15*^ zebrafish model, which showed a late onset retinal degeneration associated with loss of visual function, characterised using both *in vivo* and *ex vivo* assessment. The adult onset disease in these fish allowed us to investigate the retinal events that preceded the widespread degeneration, uncovering insightful changes within the retina and RPE that support the human clinical phenotype.

The RPE plays an essential role in the maintenance of photoreceptors, exchanging nutrients, ions and waste products, phagocytosing shed outer segments and recycling photopigment for the visual cycle^8^. Furthermore, melanosomes in the RPE serve to reduce harmful back-scattered light and remove free radicals produced during these processes. Electron microscopy of the *obe*^*td15*^ zebrafish RPE revealed changes to the phagosome clearance, mitochondrial number and melanosome movement not previously described together. In fish and amphibians, the melanosomes of the RPE exhibit a dramatic redistribution from the basal cell body into the apical processes upon the onset of light, which is reversed in the dark^21^. Melanosomes in the mammalian RPE also show modest movement during the light cycle^25^. We found that in dark-adapted *obe*^*td15*^ fish, the melanosomes were shown to have a more basal aggregation within the RPE cells, indicating an increased motility of these mutant organelles in the absence of light. This relates to previous findings that Kir7.1 is involved in regulating melanosome distribution in the melanophores of the zebrafish skin, resulting in a distinct stripe pattern^19^. Defects in melanosome movement have been associated with several human retinal diseases, such as Usher syndrome^26^ and choroideremia^27^ and this may also be a contributing factor to retinal dysfunction in *obe*^*td15*^ zebrafish and patients with *KCNJ13* mutations.

Phagosome clearance was a novel process found to be affected in the *obe*^*td15*^ zebrafish RPE. Daily, the RPE phagocytoses portions of the photoreceptor outer segments and the resulting phagosomes are required to move from the apical to the basal region in order to mature and acquire the capacity to fuse with lysosomes for degradation^28^. In *obe*^*td15*^ fish at 3 and 6 mpf, there was an increased accumulation of phagosomes in the RPE cells, suggesting that phagosome processing may be disrupted. As the Kir7.1 expression has been localized to the apical RPE^5^, it is feasible that this protein may play a part in the process. In mice, the Kir7.1 protein has been found to interact with Rab28 and may facilitate this protein in cone outer segment phagocytosis^29^. Defects in phagocytosis and phagosome degradation by the RPE are associated with several cellular metabolic outcomes, such as lipid accumulation, oxidative stress and mitochondrial dysfunction^30–33^, and have been found in models of other retinal diseases including age-related macular degeneration^34,35^, Usher syndrome^36^ and choroideremia^27,37^. Considering that significant alterations in phagosome clearance were seen by 3 mpf, preceding other notable mitochondrial changes, we propose that the retinal disease in this model is primarily a failure of RPE phagosome physiology with a secondary mitochondrial dysfunction. Phagosome abundance was similar to WT at 12 mpf, which may reflect a shift in disease stage from compensatory to degenerative.

One of the most striking changes we observed in the *obe*^*td15*^ RPE was the vast mitochondrial expansion noted at 6 and 12 mpf, in which significant increases in both mitochondrial number and size were found along with upregulation of *polg2*, an indicator of mitochondrial number, and several other mitochondria-related genes. The outer retina has the greatest metabolic demand in the body, owing largely to the photoreceptor activity, and the RPE has an enriched mitochondrial population to meet the high-energy needs of these cells to ensure function and survival^38^. The main role of mitochondria is to provide energy in the form of ATP, in addition to numerous other tasks which include regulation of apoptosis stress response pathways and calcium levels. These organelles are not static and are known to respond to the energetic needs of their environment. The observed changes in mitochondrial biogenesis in the mutant RPE may be a compensatory response to unmet energy demands or retinal stress caused by Kir7.1 channel dysfunction. The reduction in ATP levels and OCR may also suggest an impairment of mitochondrial function in the mutant retina as there is insufficient ATP production despite their increased numbers. Kir7.1 is thought to restore sub-retinal K^+^ levels essential for proper visual function and is coupled with the Na^+^/K^+^-ATPase pump^12–14^; in the circumstance of Kir7.1 dysfunction, altered pump activity may deplete ATP. Alternatively, the mitochondrial changes could be related to ER stress resulting from retention of mutant Kir7.1 proteins in the cytosol; however, Iwashita *et al*. (2006) found that the mutant Kir7.1 channel was still able localize to the cell membrane although K^+^ conductance was abolished^19^. We investigated GRP78 expression as a marker of ER stress, but found no evidence of this in the *obe*^*td15*^ retina (data not shown).

In addition to their abundance, mitochondria in the *obe*^*td15*^ RPE were greater in size than WT from 6 mpf onwards. In *obe*^*td15*^ retina, the electron-lucent mitochondria found in the apical region of the red-green double cone inner segment ellipsoid were also enlarged. Mega-mitochondria, which are large electron-dense mitochondria with higher ATP production, have been previously described in this photoreceptor region, which increase in size independently of mitochondrial fusion during development; these were present in both WT and *obe*^*td15*^ cones^39,40^. However, the electron-dense mitochondria in the *obe*^*td15*^ fish displayed juxtaposed linearized cristae membranes. This feature has been described in skeletal muscle mitochondria of patients that have a mtDNA mutation (*m.8344A* > *G)*, and has been proposed to be due to changes in the membrane lipid composition^41^. As ATP levels were reduced despite the mitochondrial enlargement in the *obe*^*td15*^ retina, the alterations are likely indicative of dysfunction.

In several systems, it has been shown that mitochondrial morphology can show changes in size, shape and membrane organization as a result of ageing or stress. Enlarged mitochondria are typically a sign of pathology in dysfunctional cells^42^, including muscle^43,44^, cardiomyopathy^45^ and by forced senescence in culture^46^. In the murine RPE, metabolic or oxidative stress resulted in the formation of large mitochondria produced by activation of the P13/AKT/mTor signaling pathway^47^. From our data, a decrease in mitochondrial size with age appears to be the normal progression in WT zebrafish, which has also been observed in human RPE cells^48,49^. To explore whether the abnormal growth in the *obe*^*td15*^ retina may be related to alterations in the mitochondrial dynamics, we investigated *fis1* and *opa1* expression as indicators of fission and fusion, respectively. Mitochondria are able to use these processes in response to their environment^50^. Fusion supports mitochondrial function by mixing contents of partially damaged mitochondria and is stimulated by energy demand and stress, while fission creates new mitochondria and removes those that are damaged. *opa1* and *fis1* were both upregulated at 6 mpf in the mutant retina, albeit significantly for *opa1* only, which may reflect an attempt to compensate for elevated stress and energy demands at this time point and may also contribute to the observed morphological changes. Large mitochondria are autophagocytosed less readily than small ones^43^, also potentially causing oversized organelles to accumulate in the mutant. Further experimentation will be necessary to explore these ultrastructural changes. If mitochondrial abnormalities contribute to the *KCNJ13* retinopathy, treatments that would support mitochondrial function e.g near infrared light therapy^29^ may be promising therapeutic strategies.

As the mitochondrial alterations in the mutant retina were indicative of early stress events preceding degeneration, we examined expression of several genes and proteins associated with stress response pathways. At 6 mpf, activation of the Müller cells became apparent through increased expression of GFAP through their retinal processes, a characteristic sign of retinal injury or stress^22^. Depleted ATP levels, also noted at 6 mpf, are associated with metabolic and oxidative stress and even moderate reductions have been found to significantly contribute to oxidative stress in RPE cells^33,51^. hsp60 is a highly conserved mitochondrial chaperone that assists protein folding and facilitates proteolytic degradation of denatured proteins and is typically upregulated in response to mitochondrial stress^24^. We found that hsp60 expression was robustly increased at 6 and 12 mpf in the *obe*^*td15*^ retina. Previously, a premature decline in ATP associated with changes in hsp60 expression was found in the *Cfh*^−/−^ mouse model of retinal degeneration, preceding phenotypic changes^52^. We investigated expression of an additional stress-related gene *bcl2a*, which was found to be significantly upregulated at 6 mpf in the *obe*^*td15*^ retina. This gene is orthologous to *BCL2*, an apoptosis suppressor known to facilitate mitochondrial DNA repair and support cell survival^53^. Overexpressing *BCL2* or using compounds that cause its upregulation has been shown to inhibit retinal cell death in several rodent models^54–58^ and cellular models^53,59,60^. Increased expression at 6 mpf in the mutant retina may be a protective response to a stressed environment, inhibiting apoptosis and contributing to the preservation of retinal structure at this time point.

To investigate oxidative stress, we examined expression of antioxidant enzymes *sod1* and *sod2* as indicators of reactive oxygen species (ROS) levels^61^, which were found to be upregulated at 12 mpf in the *obe*^*td15*^ retina. ROS are generated as by-products of the electron transfer chain and build up can compromise mitochondrial function by damaging their DNA, implicating mitochondria as both generators and targets of oxidative stress. Elevated levels of ROS in the retina can be induced by environmental factors such as blue light^62^ and are associated with ageing and disease^63^. Increased expression of the superoxide enzymes genes in the *obe*^*td15*^ retina may be a consequence of prolonged mitochondrial dysfunction causing an accumulation of ROS, which may in part trigger widespread degeneration. Additionally, at 12 mpf we observed a downregulation of *bcl2a* expression, which has been previously reported in oxidative stress-associated retinal cell death^64–67^. This evidence suggests that oxidative stress plays a role in the Kir7.1 pathophysiology.

Previously, Kir7.1 channel function has been examined by patch-clamp recording of CHO-K1 cells expressing WT or mutant human Kir7.1^10^, and isolated mouse RPE cells^14^. In addition, ERG responses have been recorded from mouse retinas where Kir7.1 was inhibited^10,14^ or mutated^17,18^, with relatively short-term examination of the retina (≤3 months). Altogether, these studies have demonstrated the essential contribution of Kir7.1 to RPE potassium conductance and ERG physiology. In our study, we have used longitudinal assessment to investigate the disease natural history and identify potential novel functions for the Kir7.1 protein in RPE. For future investigations, we will also aim to use similar techniques to those employed previously to explore the Kir7.1-related electrophysiological defects in the zebrafish retina to further examine channel activity in zebrafish and ensure functional conservation between species.

In summary, in-depth longitudinal analysis of the *obe*^*td15*^ zebrafish retina has been valuable in providing insight into Kir7.1 dysfunction, which manifests as alterations in the activity of phagosomes and mitochondria in the RPE and photoreceptors. These changes precede retinal degeneration, highlighting these organelles as potential novel therapeutic targets for *KCNJ13*-related disease. Establishing whether similar changes are seen in patient cells harbouring *KCNJ13* mutations will be an essential next step.

## Materials and Methods

### Zebrafish husbandry

Zebrafish (wild-type, AB-strain [WT]) and *obe*^*td15*^ zebrafish were bred and maintained according to local UCL and UK Home Office regulations for the care and use of laboratory animals under the Animals Scientific Procedures Act at the UCL Institute of Ophthalmology animal facility. UCL Animal Welfare and Ethical Review Body approved all procedures for experimental protocols, in addition to the UK Home Office (License no. PPL PC916FDE7). All approved standard protocols followed the guidelines of the ARVO Statement for the Use of Animals in Ophthalmic and Vision Research Ethics^68^.

WT and *obe*^*td15*^ zebrafish were generated by natural pair-wise matings of genotyped homozygous fish and raised at 28.5 °C on a 14 h light/10 h dark cycle in the UCL zebrafish facility. Heterozygous *obe*^*td15*^ *z*ebrafish were not examined in this study. At given timepoints, namely 3, 6 and 12 months post fertilization (mpf), WT and *obe*^*td15*^ *z*ebrafish were terminally anaesthetized in 0.2 mg/ml Tricaine (MS-222) and the eyes were harvested through enucleation.

### Wholemount morphology and retinal histology

Wholemount zebrafish were imaged using a Nikon SM-1500 stereomicroscope with a Nikon digital sight DS-Fi2 system. Enucleated eyes fixed in 4% paraformaldehyde/PBS at 4 °C overnight before processing and embedding using the JB-4 embedding kit (Polysciences Inc.) with 7 µm thick sections as described^69^. Sections were imaged using a Leica DMRB with Jenoptik D-07739 Optical System.

### RNAscope assay

WT zebrafish eyes (~6 mpf) were enucleated and fixed in 4% paraformaldehyde/PBS at 4 °C overnight. After washing 3 times in PBS for 10 minutes, the eyes were incubated in 10%, 20% and 30% sucrose/PBS at 4 °C overnight each time. The eyes were frozen embedded in Tissue-Tek O.C.T embedding medium (VWR) using dry ice and 14 um sections were collected onto Superfrost PLUS slides (VWR) using a Leica CM1850 cryostat. Tissue was washed with 1X PBS for 5 minutes to remove O.C.T, followed by boiling in RNAscope Target Retrieval reagent (Advanced Cell Diagnostics, ACD) for 5 minutes. Afterwards, slides were briefly washed with sterile water and incubated for 15 minutes at 40 °C with RNAscope Protease III reagent (ACD). Fluorescent *in situ* hybridization staining was performed using the RNAscope Fluorescent Multiplex Detection kit (ACD) according to the user’s manual. The *kcnj13* target probe and *odc1* and *dapB* control probes were designed and provided by ACD. Slides were mounted in Prolong Gold Antifade mountant (Thermo Fisher) and imaged using a Leica LSM 710 confocal microscope.

### Optical coherence tomography (OCT)

WT and *obe*^*td15*^ retinas were scanned using the Bioptigen Envisu R2200 Spectral Domain Ophthalmic Imaging System (Bioptigen, Inc.) as described^20^. Retinal thickness (ganglion cell layer to RPE) was measured using the Bioptigen InVivoVue Software calipers. The regularity of the cone photoreceptor mosaics was analyzed using Voronoi domain analysis to provide a statistical assessment of orderliness. To do this, photoreceptor cells were manually identified using ImageJ (National Institute of Health) from *en face* OCT scans which were extracted from the volume. Cell coordinates were analyzed using ‘Voronoi’ function in MATLAB (MathWorks). The percentage of six-sided cells and distribution of sidedness were assessed.

### Optokinetic response analysis

WT and *obe*^*td15*^ zebrafish were lightly anaesthetized in 0.2 mg/ml tricaine and placed in a custom-made foam holder supported by dissection pins in a 55 mm petri dish. The dish was filled with tank water and the fish were allowed to regain consciousness, then placed into a custom-made optokinetic device consisting of a 12 cm acrylic drum, rpm adjustable rotating motor with laser tachometer and stereo microscope (Zeiss Stemi-2000C) c-mounted with a digital SLR camera (Nikon D5100). Each zebrafish was assessed with varying grating lengths (from 0.4 cm to 0.04 cm in 0.04 cm increments) at a consistent rpm speed (12 rpm) until the stripes could no longer be tracked by the zebrafish, following published protocols^70^. Visual acuity was calculated as cycles per degree (cpd) using the following equation:

$$\frac{1}{2ta{n}^{-1}(\frac{h}{2a})}$$

where *a* is the distance from the center of the lens to the grating, and *h* is the length of one cycle of the smallest grating at which an optokinetic response was observed. As a positive control, three WT zebrafish at 6 mpf received an intravitreal injection of 0.1 ml of 10 µm ouabain to induce a chemical retinal degeneration, and were assessed at day 3 post-injection, a stage at which the retina is known to be ablated^71^.

### Transmission electron microscopy (TEM)

All fish were fully light-adapted upon collection (at midday) unless being dark-adapted, in which case they were incubated without light for 48 hours before collection. Enucleated eyes from WT and *obe*^*td15*^ *z*ebrafish were fixed and embedded as previously described^72^. Using a Leica EM UC7 ultramicrotome, 100 nm sections were cut, collected on copper grids (EMS) and stained with lead citrate (−). Sections were examined on a Jeol 1010 TEM and imaged using a Gatan Orius SC1000B charge-coupled device camera. Images were analyzed using ImageJ software.

### Immunostaining of retinal cryosections

Fresh enucleated eyes were fixed with 4% paraformaldehyde/PBS overnight at 4 °C before incubation in 30% sucrose/PBS for 6 hours at room temperature. The samples were mounted and frozen in TissueTek O.C.T. (VWR) using dry ice. 14 µm sections were cut using a Leica CM1850 cryostat and collected onto Superfrost PLUS slides (VWR). Sections were then washed in PBS/0.1% Triton-X (Sigma) before being blocked for 1 hour with 20% normal goat serum (Sigma-Aldrich) in PBS/0.1% Triton-X and then incubated in rabbit anti-ZRF1 (ZDB-ATB-081002-46, ZIRC) diluted 1:500 in antibody solution (1% normal goat serum in PBS/0.5% Triton-X) at 4 °C overnight. After washing three times with PBS/0.5% Triton-X, the sections were incubated in secondary Alexa Fluor 647 antibody (Thermo Fisher) diluted 1:500 in antibody solution for 2 hours at room temperature. Finally, the sections were counterstained and mounted using Prolong Diamond Antifade mountant + DAPI (Thermo Fisher). The slides were imaged using a Leica LSM 710 confocal microscope.

### Quantitative (q)RT-PCR

RPE and retinal tissue was isolated from enucleated WT and *obe*^*td15*^ zebrafish eyes. Total RNA was extracted using the RNeasy micro kit (Qiagen, UK) according to the manufacturer’s instructions. Using 500 ng total RNA, cDNA was reverse transcribed using the Superscript III First-strand synthesis Supermix kit (Thermo Fisher). For quantitative real-time PCR amplifications, gene expression was quantified using SYBR Select fluorescent dye (Thermo Fisher) in triplicate reactions for each sample. All qRT-PCR primers are listed in Table 1. The expression of each gene was normalized to the geometric mean of *β-actin*, *ribonucleoprotein L13a* and *ef1α* internal housekeeping genes. The StepOne Plus RealTime PCR System (Thermo Fisher) was used and reactions analyzed using the Comparative CT experiment option in the StepOne software (Version 2.3).

**Table 1:** Genotyping PCR and qRT-PCR primers sequences.

Gene	Forward primer (5′-3′)	Reverse primer (5′-3′)
*kcnj13*	TCATCCTAATTTCCTCTCCACC	TGTCGATGCTGAACTCCAGAG
*polg2*	GTGGAGGAAGTTTGCTTTAGGCCCG	GGGTCCACAGTGTCTCCAGCGT
*fis1*	ACAGACTTAAGGAGTATGAGAGAGC	AATACCACCGACAATCGCCA
*opa1*	GCCGGAAGTGTAGTTACCTG	AGGTGGTCTCTGTGGGTTGT
*sod1*	GGTGACAACACAAACGGCTG	TGGCATCAGCGGTCACATTA
*sod2*	ACAGCAAGCACCATGCAACA	CAGCTCACCCTGTGGTTCTCC
*bcl2a*	GAGGTTGGGATGCCTTCGTG	CCAAGCCGAGCACTTTTGTT
*β-actin*	TGTACCCTGGCATTGCTGAC	TGGAAGGTGGACAGGGAGGC
*L13a*	TCTGGAGGACTGTAAGAGGTATGC	AGACGCACAATCTTGAGAGCAG
*ef1α*	CTGGAGGCCAGCTCAAACAT	ATCAAGAAGAGTAGTACCGCTAGCATTAC

### Adenosine triphosphate (ATP) assay

ATP was measured by luciferin–luciferase assay (Enliten ATP Assay System, Promega). The retina (including RPE) was dissected from enucleated eyes of WT and *obe*^*td15*^ fish and placed in Krebs solution. The samples were transferred to 2.5% trichloroacetic acid (TCA), then homogenized by sonication (3 × 10 seconds, XL-2000 Qsonica LLC) on ice. Cell debris was removed by centrifugation at 13,000 rpm for 30 minutes at 4 °C. The supernatant was collected and the TCA was neutralized with 1 M Tris–acetate buffer (pH 7.75, final TCA concentration 0.0625%). Protein concentration was measured using the BCA Protein Assay Kit (Pierce), a plate reader (Tecan Safire) and Magellan Software. To analyze ATP levels, 10 µl of the neutralized samples was added to 100 µl of luciferin–luciferase in fresh buffer and ATP was measured using a Glomax-20/20 luminometer (Promega) and data normalized to concentration of protein.

### OCR measurement

Measurement of baseline OCR was performed using a Seahorse XFe96 analyzer (Agilent). Whole retinas were isolated from WT, *obe*^*td15*^ and ouabain-injected zebrafish and 0.75 mm punch biopsies were taken and loaded onto a 96-well plate. The retinal punches were incubated in Seahorse XF base medium (Agilent) supplemented with 12 mM glucose, 10 mM HEPES and 26 mM NaHCO_3_ to measure OCRs for 40 minutes at 28.5 °C.

### Western Blot

RPE and retinal tissue was isolated from enucleated WT and *obe*^*td15*^ zebrafish eyes and snap-frozen in liquid nitrogen. Samples were analyzed by Western blot assay as previously described^69^ using primary rabbit anti-hsp60 antibody diluted 1:1000 (Abcam) and secondary anti-rabbit IgG HRP conjugate diluted 1:10,000 (Sigma) in blocking solution (5% dry milk, PBS/0.1% Tween [PBS-T]). The membrane was stripped and re-probed with 1:5000 polyclonal anti-β-actin antibody (Sigma) as a loading control. ImageLab software (BioRad) was used to determine the relative abundance of hsp60 compared with corresponding levels of control protein expression.

### Statistics

Data are shown as mean values ± SEM from *n* observations. The Shapiro-Wilk test was used to test for normal distribution. Student’s t-tests or Mann-Whitney U tests were used to compare WT and *obe*^*td15*^ data. P < 0.05 was accepted to indicate statistical significance (*). SPSS software (IBM) was used for statistical analysis.

## Supplementary information

Supplementary Information

## Data Availability

All data generated or analysed during this study are included in this published article and (its Supplementary Information Files) or available from the corresponding author on reasonable request.
