# Supplementary material for: Phagosomal and mitochondrial alterations in RPE may contribute to KCNJ13 retinopathy
Source: Sci Rep. 2019 Mar 7;9:3793. doi: 10.1038/s41598-019-40507-8 (PMC6405871; doi:10.1038/s41598-019-40507-8)
Supplement: Supplementary file 1 — Supplementary Information [file 41598_2019_40507_MOESM1_ESM.pdf]

Supplementary Information

Phagosomal and mitochondrial alterations in RPE may contribute to *KCNJ13* retinopathy

Maria Toms[1], Thomas Burgoyne[1], Dhani Tracey-White[1], Rose Richardson[1], Adam M Dubis[1,2], Andrew R Webster[1,2], Clare Futter[1], Mariya Moosajee[1,2,3]

[1]UCL Institute of Ophthalmology, London, UK.

[2]Moorfields Eye Hospital NHS Foundation Trust, London, UK.

[3]Great Ormond Street Hospital for Children NHS Foundation Trust, London, UK.

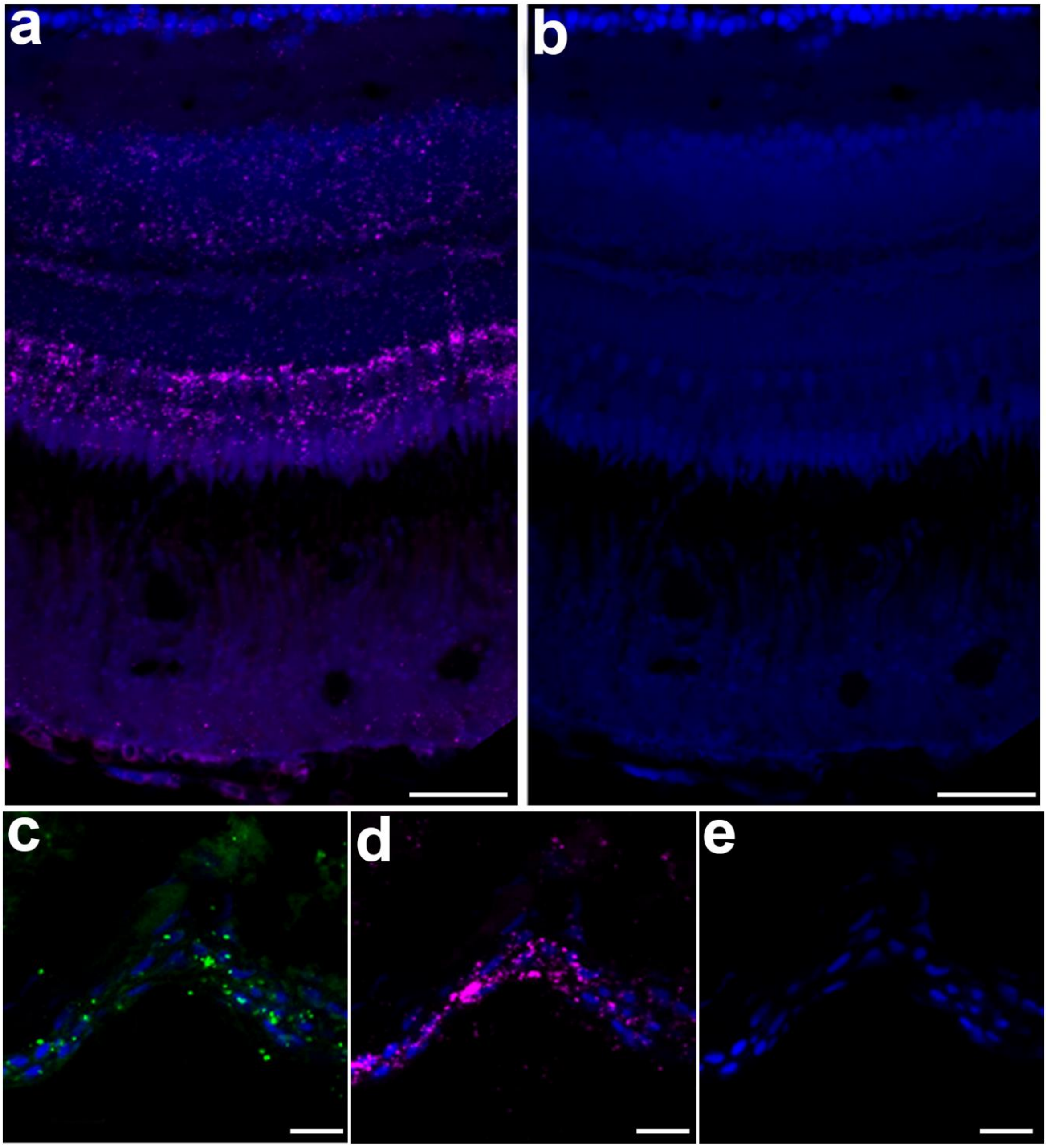

Supplementary Figure S1. RNAscope *in situ* hybridization positive and negative probes.

Wild-type adult retinal cryosections with *odc1* positive control probe (magenta) (a) and *dapB* (bacterial gene) negative control probe (b). Wild-type adult tail fin cryosections with *kcnj13* (green) (c), *odc1* (d) and *dapB* (e) probes. Sections are counterstained with DAPI (blue). Scale bars = 25 µm (a-b), 10 µm (c-e).

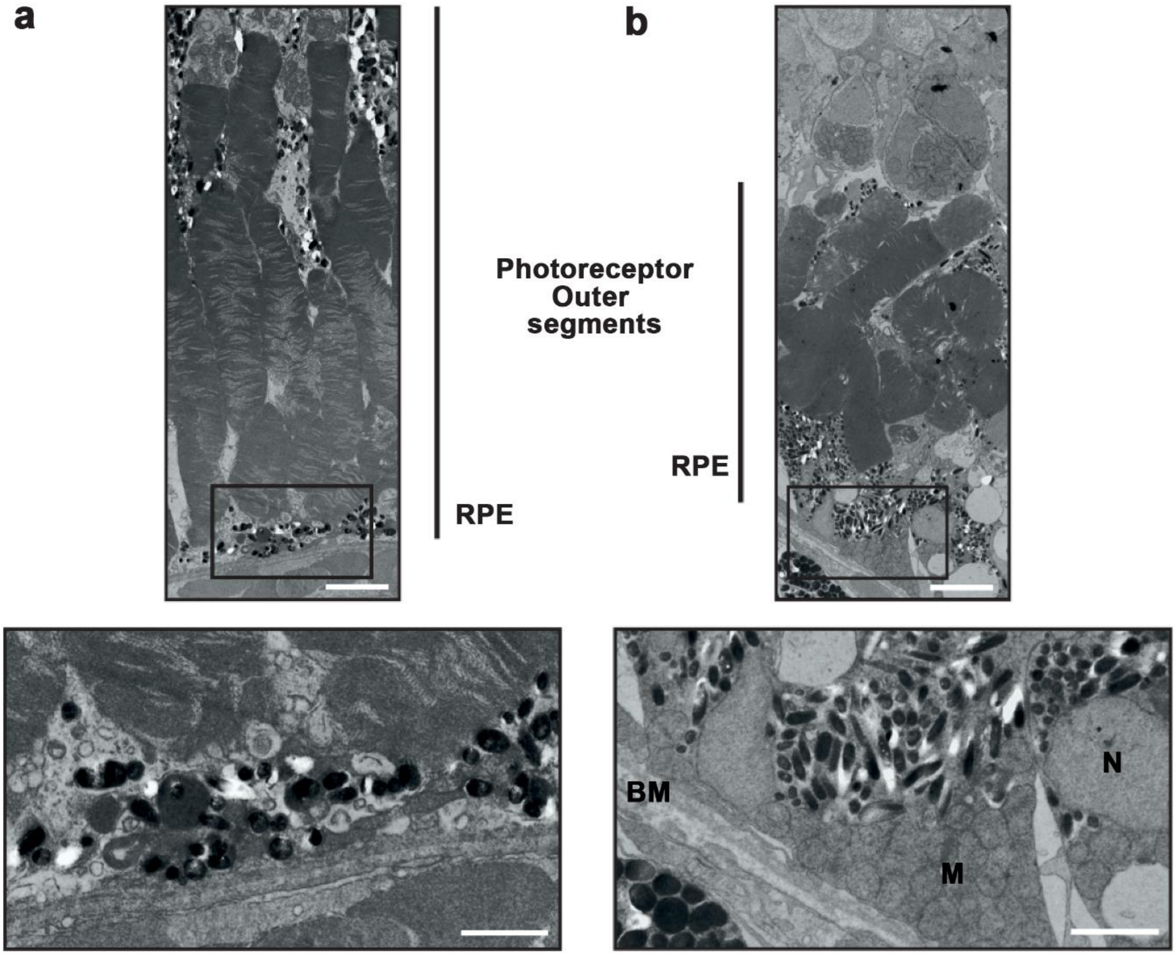

Supplementary Figure S2. *obe*^{td15} zebrafish eyes exhibit areas of photoreceptor loss with islands of preserved retina.

TEM images (a-b) of the photoreceptor outer segment and retinal pigment epithelium (RPE) from a 12 months post-fertilization *obe*^{td15} zebrafish eyes. A region displaying preserved retinal tissue (a) and an affected region (b) with disordered photoreceptor outer segments and numerous, large mitochondria in the RPE. BM, basement membrane; M, mitochondria; N, nucleus. Scale bars = 5 µm and 2 µm.

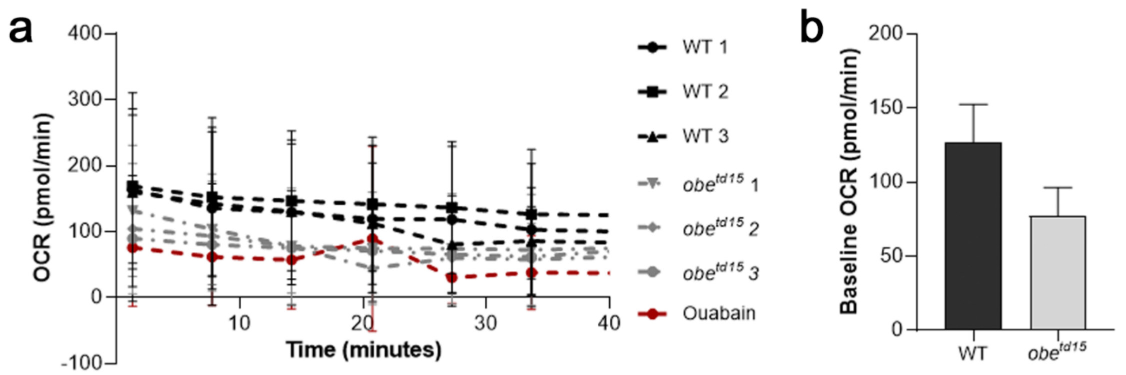

Supplementary Figure S3. Measurement of mitochondrial function using Seahorse XF analysis.

(a) Oxygen consumption rate (OCR) of 6 months post fertilization wild-type, *obe*^{td15} and ouabain-injected (4 days post injection) retinal samples (0.75 mm punch biopsies). n=6 per fish (3 punches taken per retina), mean ±SD. Baseline OCR is plotted on bar chart (b), p=0.0558. n=3, mean ±SD.

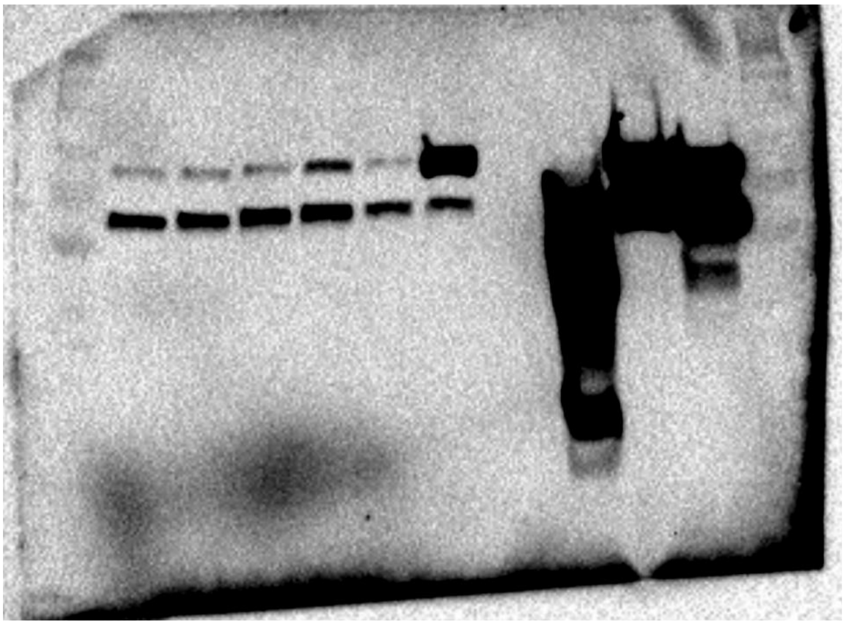

Supplementary Figure S4. Uncropped Western blot for hsp60
